# Altered Glucose Homeostasis and Hepatic Function in Obese Mice Deficient for Both Kinin Receptor Genes

**DOI:** 10.1371/journal.pone.0040573

**Published:** 2012-07-19

**Authors:** Carlos C. Barros, Anderson Haro, Fernanda J. V. P. Russo, Ines Schadock, Sandro S. Almeida, Rosane A. Ribeiro, Emerielle C. Vanzela, Valeria P. Lanzoni, Flavio C. Barros, Milton R. Moraes, Marcelo A. Mori, Reury F. P. Bacurau, Martin Wurtele, Antônio C. Boschero, Everardo M. Carneiro, Michael Bader, Joao B. Pesquero, Ronaldo C. Araujo

**Affiliations:** 1 Department of Biophysics, Federal University of São Paulo, São Paulo, Brazil; 2 Max-Delbrück-Center for Molecular Medicine, Berlin, Germany; 3 State University of Campinas, Campinas, Brazil; 4 Paulista University, São Paulo, Brazil; 5 School of Arts, Sciences and Humanities, University of São Paulo, São Paulo, Brazil; 6 Department of Science and Technology, Federal University of São Paulo, São José dos Campos, Brazil; 7 Department of Pathology, Federal University of São Paulo, São Paulo, Brazil; Universidade de Sao Paulo, Brazil

## Abstract

The Kallikrein-Kinin System (KKS) has been implicated in several aspects of metabolism, including the regulation of glucose homeostasis and adiposity. Kinins and des-Arg-kinins are the major effectors of this system and promote their effects by binding to two different receptors, the kinin B2 and B1 receptors, respectively. To understand the influence of the KKS on the pathophysiology of obesity and type 2 diabetes (T2DM), we generated an animal model deficient for both kinin receptor genes and leptin (obB1B2KO). Six-month-old obB1B2KO mice showed increased blood glucose levels. Isolated islets of the transgenic animals were more responsive to glucose stimulation releasing greater amounts of insulin, mainly in 3-month-old mice, which was corroborated by elevated serum C-peptide concentrations. Furthermore, they presented hepatomegaly, pronounced steatosis, and increased levels of circulating transaminases. This mouse also demonstrated exacerbated gluconeogenesis during the pyruvate challenge test. The hepatic abnormalities were accompanied by changes in the gene expression of factors linked to glucose and lipid metabolisms in the liver. Thus, we conclude that kinin receptors are important for modulation of insulin secretion and for the preservation of normal glucose levels and hepatic functions in obese mice, suggesting a protective role of the KKS regarding complications associated with obesity and T2DM.

## Introduction

Obesity and type 2 diabetes mellitus (T2DM) are important public health problems in several countries [1]. Both conditions are associated and the risk for T2DM can increase by about 300% in obese subjects [2], while weight gain seems often to precede the development of T2DM. Therefore, the higher incidence of obesity in several populations in the last three decades has been accompanied by an increase in T2DM [3].

T2DM involves changes in carbohydrate, lipid and protein metabolisms and is directly linked with insulin resistance and the inability of pancreatic beta cells to compensate this phenomenon [4]. The importance of the liver in the pathophysiology of T2DM is also widely recognized. The liver is the main organ responsible for endogenous glucose production, and increased hepatic glucose production is the major cause of hyperglycemia in poorly controlled T2DM patients [5]. Additionally, it has been shown in obese subjects that fatty livers increase insulin resistance and promote T2DM [6].

Non-alcoholic fatty liver disease (NAFLD) is a term used to describe a wide spectrum of liver damages, ranging from simple steatosis to steatohepatitis and cirrhosis. NAFLD is associated with obesity and insulin resistance [7]. Non-alcoholic steatohepatitis (NASH), a subtype of NAFLD characterized by hepatocyte injury and inflammation leading to increased risk of cirrhosis development, is present in 10% of patients with T2DM [8].

Kinins are vasoactive peptides locally produced in the circulatory system and other tissues by the action of serine proteases called kallikreins. Bradykinin (BK) and des-Arg^9^-bradykinin (Des-Arg-BK) are the main effectors of the KKS in rodents. These peptides bind to the B2 and B1 kinin receptor, respectively [9], leading to the sensation of pain, vasodilatation, vascular permeability, inflammation and oedema formation [10]. These receptors have also been linked to the pathophysiology of diabetes [11], [12]. BK increases glucose uptake in muscle and adipose tissue [13] while the BK metabolite Des-Arg-BK has been shown to control leptin sensitivity in the hypothalamus [14] and insulin release in pancreatic islets [15].

To study the involvement of the KKS in the pathophysiology of T2DM in the context of obesity, we generated genetically obese mice (*ob/ob*) lacking both kinin B1 and B2 receptors. We were thus able to show that complete abrogation of kinin function has dramatic consequences on metabolism, which includes exacerbation of NAFLD and impairment of glucose homeostasis and pancreatic function in obese mice.

## Results

### ObB1B2KO Mice Present Similar Body Mass and Body Composition when Compared to obWT Controls

**Figure 1:**
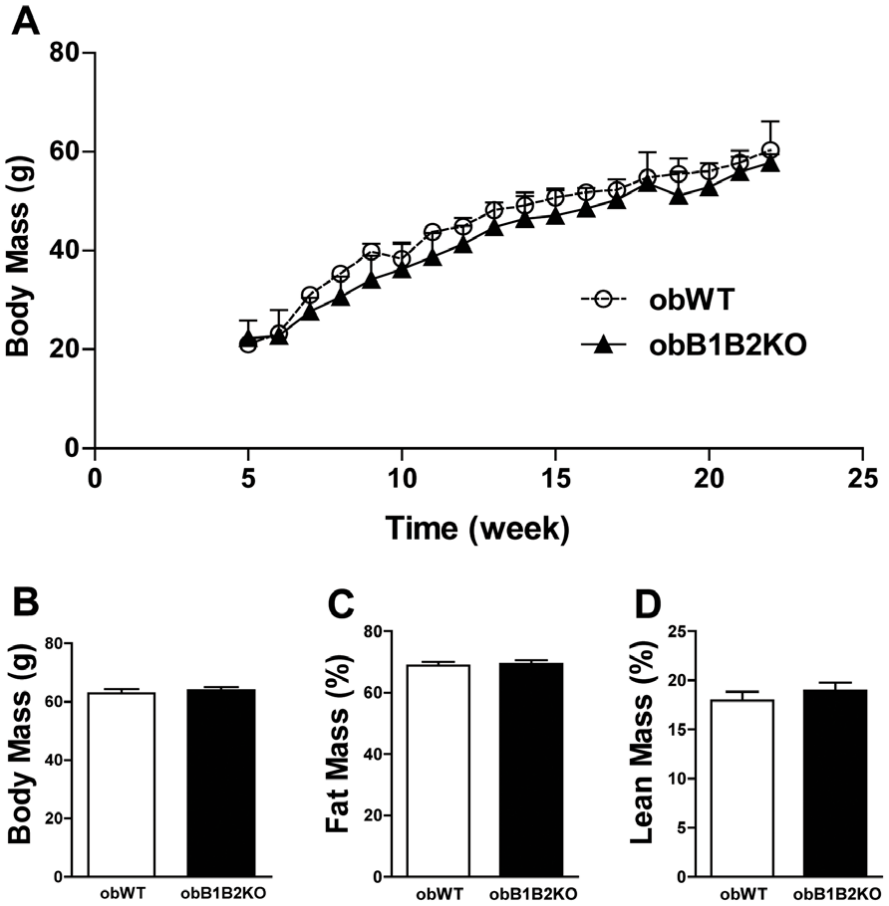
ObB1B2KO have similar body weight and body composition. A) Growth curve showing the body weight of mice from 6 to 24 weeks of age; B) Average body weight of 30-week-old mice; C and D) Densitometry of obWT and obB1B2KO at the age of 25 weeks. Data are presented as means ± SEM; n >9.

The most obvious characteristic of *ob/ob* (obWT) mice is the severe obesity that these animals develop already at younger ages. The additional absence of functional kinin B1 and B2 receptors did not alter this condition (figure 1A and B). Analyzed by densitometry, body composition did not show significant differences in the percentage of fat and lean mass (figure 1C and D). We also analyzed the mass of some organs and tissues like the spleen, kidneys, muscle triceps sural, heart and adipose tissue. The obB1B2KO mice showed however no significant mass differences of these tissues when compared to obWT mice (table S1).

### ObB1B2KO Mice Exhibit Age-associated Hyperglycemia and Insulin Resistance

**Figure 2:**
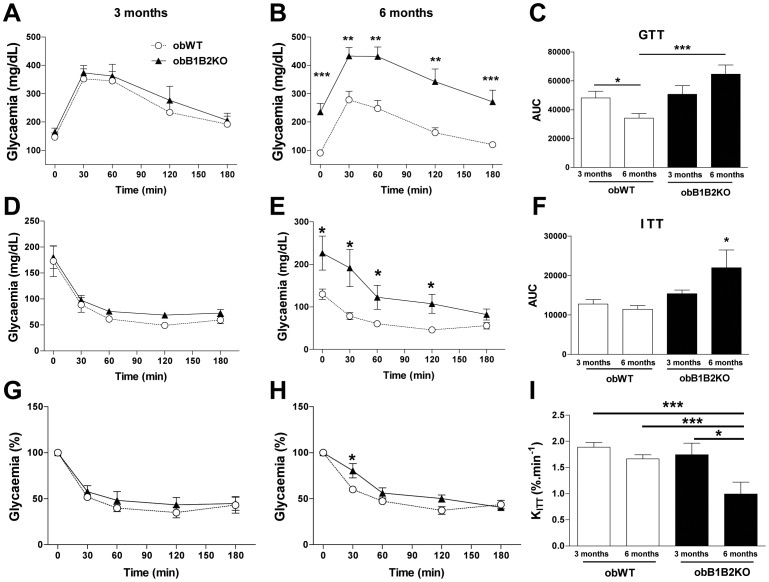
Six-month-old obB1B2KO mice present higher glycemia and insulin resistance when compared to obWT. A to C) Glucose tolerance test (GTT) of transgenic mice and controls; D to H) Absolute and relative values of the insulin tolerance test (ITT) of transgenic mice and controls. I) The constant rate of glucose disappearance (Kitt) calculated from “G” and “H”. Data are presented as means ± SEM. Student’s t-test: *, p<0.05; ***, p<0.001; n = 8.

It is known that obWT mice have improved glycemic values when growing older than 20 weeks of age [16]. This characteristic is obliterated in the obB1B2KO mice (figure 2). Three-month-old animals exhibited similar glucose and insulin tolerance (figure 2A, D and G). However, while 6-month-old obWT mice showed improved glucose tolerance and decreased glucose levels in comparison to their younger counterparts, 6-month-old obB1B2KO mice displayed a further impairment in glucose tolerance and a significant increase of fasting glycemia (Figure 2B and C). This results in drastic differences in glucose homeostasis in 6-month-old obB1B2KO when compared with obWT (figure 2). We also analyzed the insulin sensitivity in both ages (figure 2D to I). The figures 2D to 2F show that 6-month-old obB1B2KO mice present an increase of glycemia with higher area under the curve and this effect is mainly due to the differences observed in basal levels of fasting glucose. We also present the normalized data (figure 2G and H) and estimated the constant rate for glucose disappearance (K_ITT_) shown in figure 2I. The K_ITT_ analysis shows a reduction of insulin sensitivity in 6-month-old obB1B2KO mice when compared with the other groups.

### Three-month-old obB1B2KO Mice Presented Increased Hepatic Insulin Resistance

**Figure 3:**
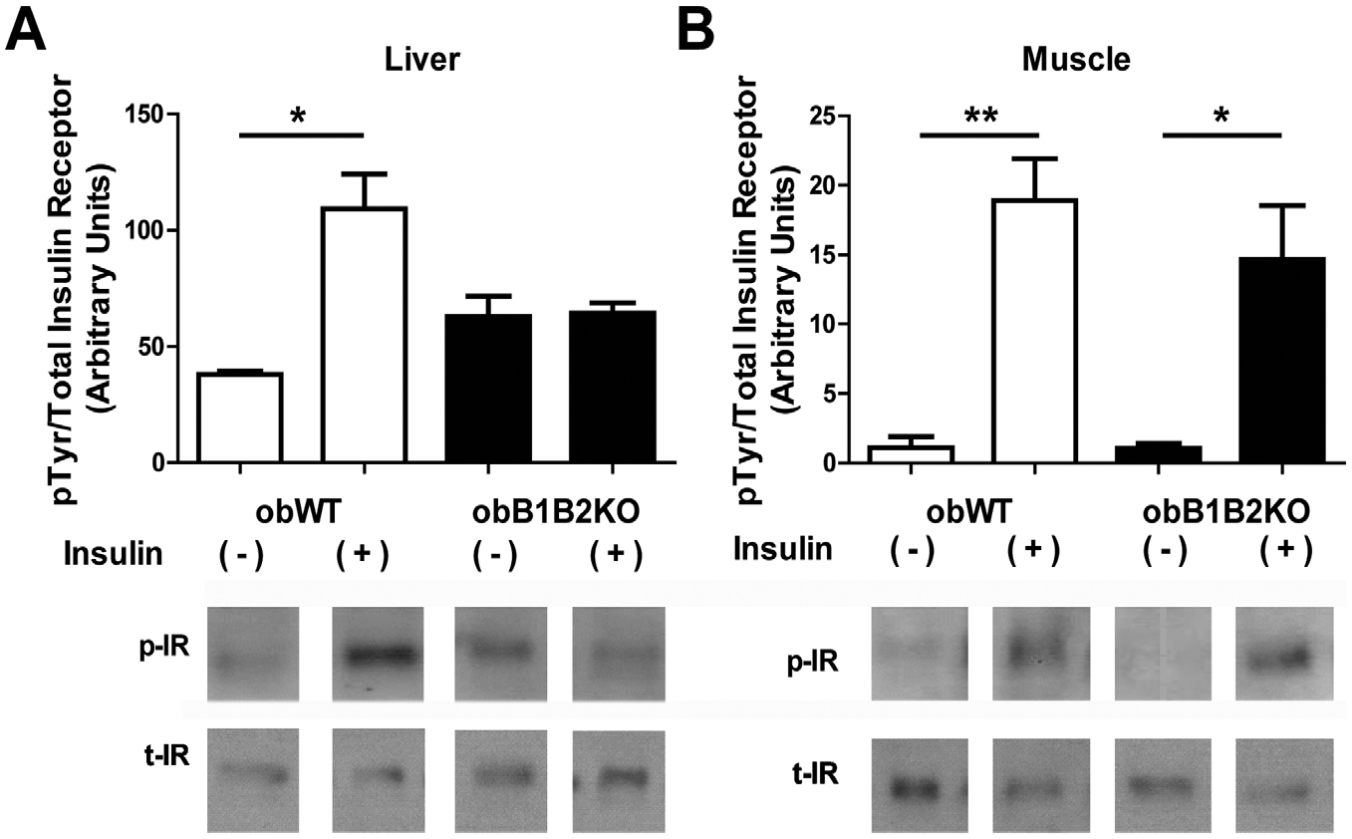
Western blot analysis of liver and muscle insulin receptor phosphorylation. Graphic and representative images of western blot analysis of phosphorylated insulin receptors (p-IR)/total insulin receptors (t-IR) of protein extracts from A) liver and B) muscle, after immunoprecipitation. Mice were starved for 12 hours overnight. After anesthesia with sodium thiopental, the abdomen was opened and insulin (+) or saline (−) were injected in the cava vein. Liver and muscle samples were collected 2 or 5 minutes after the injections. The data are presented as means ± SEM. *, p<0.05; **, p<0.01.

After immunopreciptation, western blot analysis showed inhibition of insulin receptor phosphorylation in the liver of obB1B2KO mice (figure 3A) when compared with obWT mice. On the other hand, the activation of the insulin receptor in the muscle of obB1B2KO mice is similar in obese controls (figure 3B). These results suggest the development of local insulin resistance starting in the liver.

### The Kallikrein-kinin System Modulates Insulin Secretion of Pancreatic Islets

**Figure 4:**
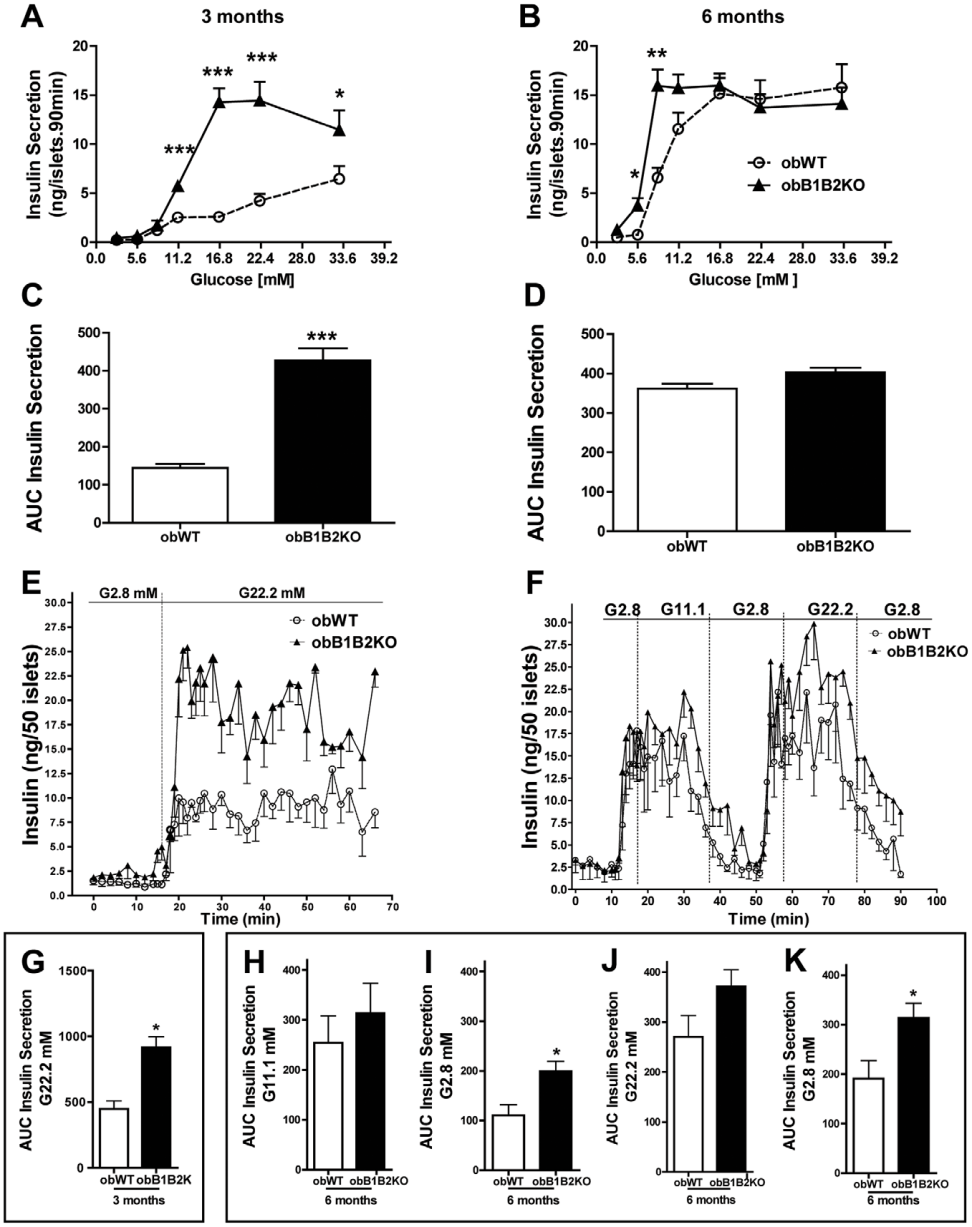
Islets from obB1B2KO mice release more insulin. Insulin secretion and analysis of pancreatic islets from 3-month-old (A and C) and 6-month-old mice (B and D) when exposed to medium containing various concentrations of glucose (EC50 = 16.97+/−0.352 and 11.30+/−0.196; for 3-month-old obWT and obB1B2KO respectively (average +/− SD) and 8.41+/−0.217 and 6.29+/−0.651 for 6-month-old mice); E and G) Dynamic analysis of insulin secretion from islets of 3-month-old mice; F and H to K) Dynamic analyzes of insulin secretion from islets from 6-month-old mice. K and I) Analysis showing extended recovery time of basal insulin secretion levels from islets of obB1B2KO mice. Data present means ± SEM. *, p<0.05; n = 8×5 islets for static analyzes and n = 4×50 islets for dynamic analyzes.

To assess how pancreatic islets could compensate for glucose intolerance in obWT mice, pancreatic islets from 3- and 6-month-old mice were isolated and incubated with different concentrations of glucose. Islets obtained from 3-month-old obB1B2KO mice secreted similar basal levels of insulin when compared to islets from obWT mice, but reacted more potently to glucose (3- to 7-fold, figure 4A and C). On the other hand, while glucose-stimulated insulin secretion increased substantially with age in islets of obWT mice, this increase was marginally observed in obB1B2KO mice (figure 4B and D). We also estimated the EC50 (effective concentration) values. At 3 months of age the EC50 glucose values were 16.97+/−0.352 mM and 11.30+/−0.196 mM for obWT and obB1B2KO respectively (average +/− SD). The EC50 values were reduced in both groups at 6 months of age and the difference between obWT and obB1B2KO were significantly reduced: 8.41+/−0.217 mM and 6.29+/−0.651 mM. To confirm these results, dynamic analysis of insulin secretion was performed.

To this end, islets were placed in mini chambers and perifused with Krebs solution containing different concentrations of glucose. Young obB1B2KO mice islets showed a higher response to glucose (figure 4E), which was not observed in older animals (figure 4F). Although no significant differences in the amount of secreted insulin during high glucose stimulation were observed in islets from 6-month-old mice (figure 4F, H and J), obB1B2KO islets needed a longer recovery time to establish basal insulin secretion levels when glucose concentration in the medium was lowered below 2.8 nM (figure 4F, I and K). Together these results suggest that insulin resistance in *ob/ob* mice can be compensated by increased insulin secretion, which is particularly observed in aged mice when glucose tolerance improves while insulin secretion increases. In mice lacking both kinin receptors, however, the increased insulin secretion can no further compensate the pronounced insulin resistance that accompanies aging in obB1B2KO mice, leading to severe glucose intolerance.

### Circulating C-peptide Levels are Elevated in obB1B2KO Mice

**Figure 5:**
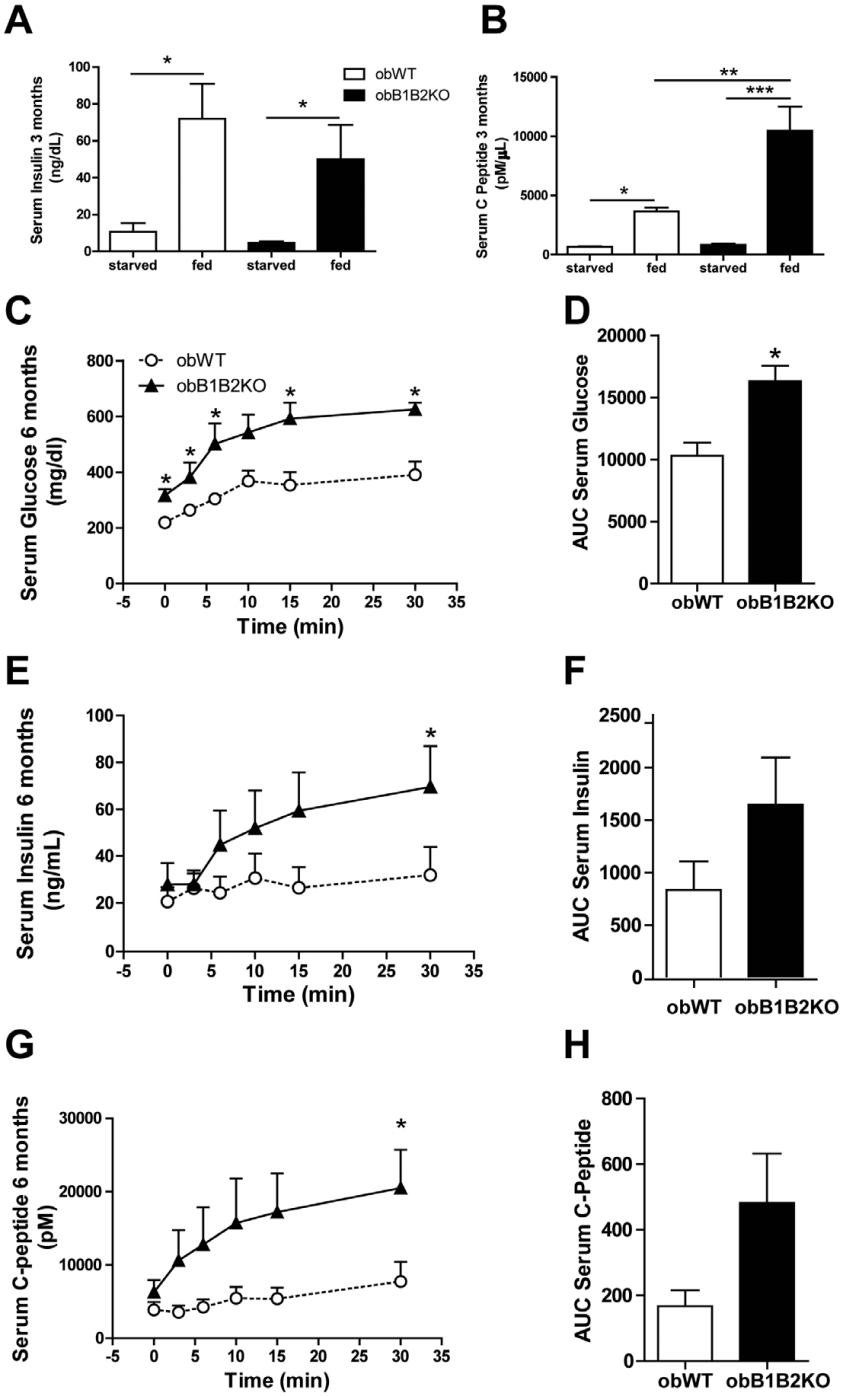
Three-month-old obB1B2KO mice have 2-fold more C-peptide levels. A) Serum insulin levels of starved and random fed mice at 3 months of age. B) Serum insulin C-peptide concentrations. C) Serum glucose after anesthesia with avertin and i.p. injection of 1 g glucose/kg body weight. D) Area under the curves of “C”. E and F) Serum insulin and, G and H) serum C-peptide from the same experiment in “C”. The data are presented as means ± SEM. *, p<0.05; **, p<0.01; ***, p<0.001, n = 8 for “A” and “B”, n = 4 for “C” to “H”.

To support the observations that younger obB1B2KO mice release higher amounts of insulin after glucose stimulation, we measured circulating insulin in blood samples taken from fasted and fed 3-month-old mice. The results obtained did not show any difference between obWT and obB1B2KO mice (figure 5A). On the other hand, insulin is a quite unstable molecule with a short half life time. Therefore, the insulin C-peptide, which is cleaved from proinsulin and released together with insulin in comparable amounts from pancreatic beta-cells, is considered a better measurement of insulin secretion *in vivo* [17]. The analysis of serum samples revealed that fed obB1B2KO mice have doubled C-peptide levels compared to their controls (figure 5B). This suggests that insulin secretion is indeed increased in younger obB1B2KO mice, but due to increased insulin clearance, measured insulin levels are comparable to those of obWT at the moment when the samples were collected.

We also measure the serum insulin and C-peptide concentrations after anesthesia and after 1 g glucose i.p. injections in 6-month-old mice. The obB1B2KO mice presented again, under these new conditions, higher glycemia (figure 5C and D). The same tendency was observed for serum insulin (figure 5E and F) and for serum C-peptide (figure 5G and H) levels.

### The Kallikrein-kinin System has a Protective Effect Against NAFLD in Obese Mice

**Figure 6:**
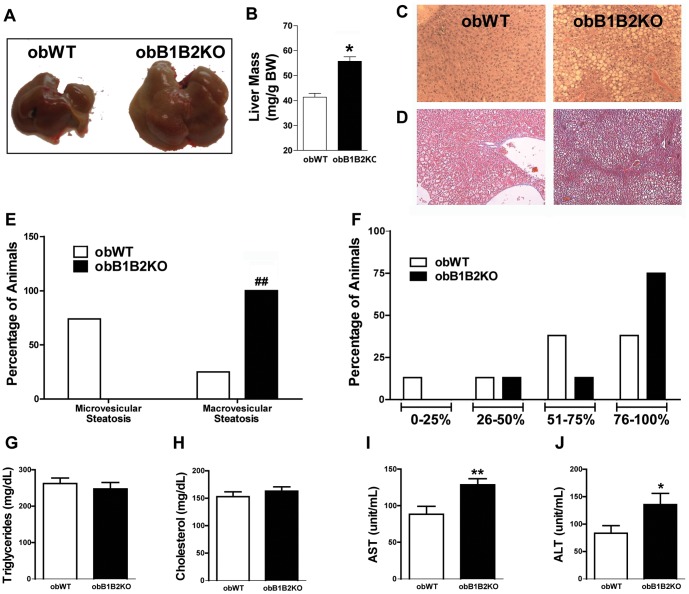
KKS protects the liver against steatosis in obese mice. A) Representative picture of the sizes of livers from 6-month-old mice; B) Averages of liver relative mass from 6-month-old mice; C) Representative picture comparing hematoxylin and eosin stained liver sections; D) Representative picture comparing Masson’s trichrome protocol; E) Percentage of the predominant type of non-alcoholic hepatic steatosis from histological analysis; F) Percentage of non-alcoholic hepatic steatosis levels from histological liver analysis; G and H) Serum lipid profile; I and J) Hepatic function analysis demonstrated by plasma transaminases measurements, aspartate aminotransferase (AST) and alanine aminotransferase (ALT). The data are presented as the means ± SEM. t student: *, p<0.05; **, p<0.01; Analysis of Contingency Table: # #, p<0.01; n = 8.

Due to the close link between NASH, obesity and T2DM, we next investigated the morphology of the liver of obB1B2KO and obWT mice. The obB1B2KO mice presented a significant increase in liver mass compared to obWT animals (figure 6A and B). The histopathological analysis showed a higher prevalence of macrovesicular lesions in the liver of obB1B2KO mice accompanied by a trend towards greater extent of areas of steatosis (figure 6C to F). We however did not find significant differences in deposition of collagen when analyzed by Masson’s trichrome staining (figure 6D). The steatotic lesions developed despite animals having similar plasma triglycerides and cholesterol levels (figure 6G and H). Additionally, we observed an increase in plasma concentrations of the aspartate aminotransferase and alanine aminotransferase transaminases, which are *bona fide* markers of liver function (figure 6I and J). These results indicate that obese mice lacking kinin receptor are more prone to liver injury.

### Six-month-old obB1B2KO Mice have Higher Gluconeogenesis and Increased Expression of Gluconeogenesis Regulatory Enzymes in the Liver

**Figure 7:**
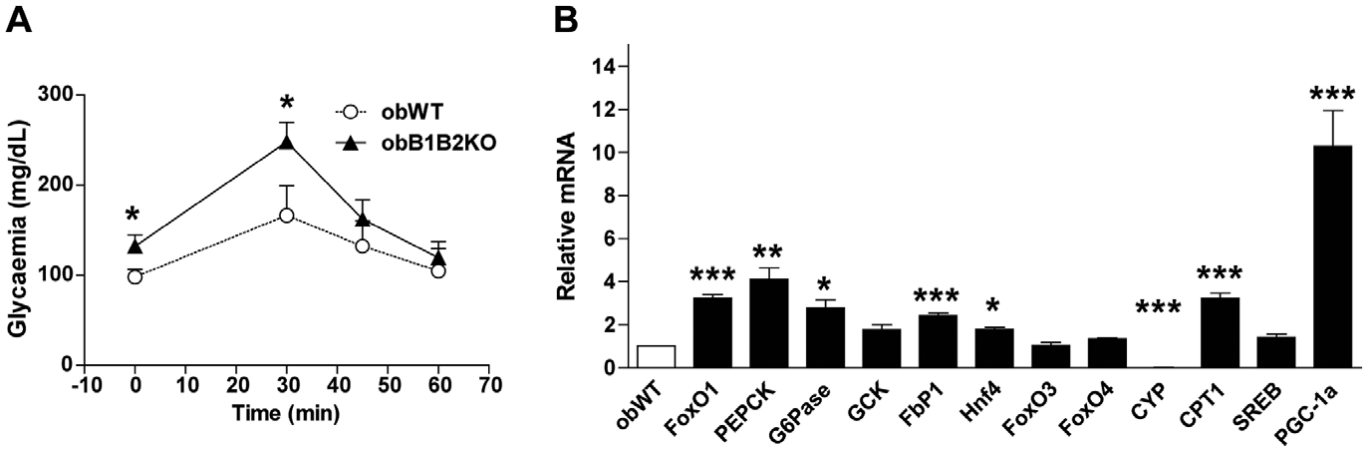
Increased gluconeogenesis and hepatic expression of FoxO1, PGC-1a and gluconeogenesis regulatory enzymes. The experiments were performed in 6-month-old mice. A) Pyruvate challenge proceeded by intraperitoneal injection of 0.5 g/kg pyruvate; B) Quantitative real time PCR: The results are expressed as changes relative to the control obWT group. Forkhead box protein O1, 03 and 04 (FoxO1, O3 and O4), phosphoenolpyruvate carboxykinase (PEPCK), glucose-6-phosphatase (G6Pase), glucokinase (GCK), fructose-1,6-bisphosphatase 1 (FbP1), hepatocyte nuclear factor 4 alpha (Hnf4), 25-hydroxycholesterol 7-alpha-hydroxylase (CYP), carnitine palmitoyltransferase-1 (CPT1), Sterol regulatory element-binding protein 1 (SREB), peroxisome proliferator-activated receptor gamma coactivator 1-alpha (PGC-1a). Data are presented as the means ± SEM. Student’s t-test: *, p<0.05; **, p<0.01; ***, p<0.001; n = 5.

The liver is the main source of glucose during fasting states and impaired hepatic gluconeogenesis is a primary cause of T2DM [5]. To test glucose production, the animals were subjected to 24 hours fasting and received an intraperitoneal injection of pyruvate. The blood glucose concentration was measured at time points 0, 30, 45 and 60 minutes after the injection. The obB1B2KO animals showed higher production of glucose after administration of pyruvate when compared to obWT mice (figure 7A).

The liver was further examined for expression of regulatory enzymes involved in the activation of gluconeogenesis: glucose-6-phosphatase (G6Pase), phosphoenolpyruvate carboxykinase (PEPCK) and fructose-1,6-bisphosphatase 1 (FbP1). Furthermore, the expression of forkhead box protein O1 (FoxO1), which regulates the expression of G6Pase and PEPCK, and peroxisome proliferator-activated receptor gamma coactivator 1-alpha (PGC-1a) were analyzed. All these factors showed increased mRNA expression in the liver of obB1B2KO compared to obWT mice, consistent with increased gluconeogenesis.

No significant differences were observed in FoxO3 and FoxO4 expression, as well as in regards to sterol regulatory element-binding protein 1 (SREB). Additionally, we observed a trend to increased expression of hepatocyte nuclear factor 4 alpha (HNF4) and a dramatic decrease of 25-hydroxycholesterol 7-alpha-hydroxylase (CYP7b1) expression, which is negatively regulated by FoxO1 [18].

## Discussion

In the present study we generated an animal model deficient in leptin and both B1 and B2 kinin receptors to study the influence of the KKS on the pathophisiology of obesity and T2DM. These animals show an age dependent increase in glycemia, insulin resistance, as well as steatosis and hepatomegaly.

These results are consistent with previous reports describing that BK can promote glucose uptake and insulin sensitivity in muscles and adipose tissues *in vitro* and in isolated systems [19], [20], [21]. These reports show increased GLUT4 translocation in these tissues when stimulated with BK. However, in the present work we show the influence of the KKS in liver metabolism, an organ that is not dependent on GLUT4. Moreover, the fact that younger animals did not show any differences in terms of glucose tolerance led us to hypothesize that other pathways that control glucose homeostasis might have also been affected in obB1B2KO mice. One obvious hypothesis was insulin secretion in pancreas. At 3 months of age, obB1B2KO mice have similar glycemia and glucose tolerance as the controls. However, increased hepatic insulin resistance was observed in 3 month-old obB1B2KO mice while the islets from obB1B2KO at the same age were able to secrete more insulin, overlapping any possible difficulty in glucose uptake caused by the KO of the kinin receptors. Nevertheless, 3-month-old obB1B2KO mice show similar insulin tolerance when compared to obWT mice, suggesting that differences in insulin resistance between these animals are age dependent and do not occur in younger mice. Thus, a defect in insulin clearance may be the most obvious explanation since insulin secretion is increased in isolated pancreatic islets from obB1B2KO mice whereas insulin levels and tolerance as well as fasting glucose levels and tolerance are similar. Although we cannot confirm this hypothesis, it is further corroborated by the observation of higher insulin C-peptide levels in 3 months old obB1B2KO mice (figure 5).

Hepatic steatosis is associated with obesity and obesity is associated with a reduced insulin clearance [22]. In this work we compared absence and presence of the two kinin receptors B1 and B2 on a leptin deficient background, showing that the degree of adiposity (figure 1), as well as the lipid profile (figure 6) are not influenced by these receptors in this model. While a large body of literature is available about insulin secretion under different pathophysiological conditions, relatively little is known about insulin clearance [22]. The liver as the main site of insulin clearance, removes approximately 50% during the first portal passage [23], [24]. Our results show that the islets of obB1B2KO mice have an increased capacity to produce insulin when stimulated with high glucose concentrations, mainly at 3 months of age. At the same time, the western blot analysis shows an increase of hepatic insulin resistance and normal insulin sensitivity in skeletal muscle. In summary, 3-month-old obB1B2KO mice present increased insulin production (figure 5 and 6), increased hepatic insulin resistance while having normal insulin sensitivity in muscle (figure 3). Together these results provide the basis of a new explanation for the normal glucose uptake in animals of this age (figure 2). Following this hypothesis, after glucose injection, the more responsive islets of obB1B2KO mice are able to deliver higher amounts of insulin, which is then not completely degraded after portal passage. The remaining insulin reaches the receptors in the muscle, which are equally sensitive to insulin in obB1B2KO and in obWT muscle at this age.

With age, in *ob/ob* mice, hyperglycemia is reduced by reasons that have not been completely elucidated [16], [25], [26]. Our data show that the improvement in glucose homeostasis in 6-month-old obWT mice is accompanied by increased insulin secretion capacity of the pancreatic islets. This is consistent with observations that the islets from obese mice are bigger at this age when compared to islets from younger obese mice [25]. ObB1B2KO mice can compensate the absence of kinin receptors through higher capacity to secrete insulin at 3 months of age, but show higher hyperglycemia at 6 months of age. This is due to the inability of their islets to further increase insulin secretion to compensate increased insulin resistance and increased hepatic glucose production. These results suggest that the dysregulation of the KKS could be associated with a higher risk of developing T2DM as aging advances. Concerning the increase in prevalence of T2DM in children and adolescents [27], [28], it is important to note that the risk of developing T2DM as well as its complications significantly increase with age [29]. Further studies will be necessary to analyze whether the KKS of patients with T2DM has its function modified in comparison to healthy subjects. The importance of the KKS to T2DM suggested by our results is reinforced by the fact that patients treated with angiotensin I-converting enzyme (an enzyme able to degrade kinins) inhibitors display protection against complications from this disease [30], [31], [32].

Our results show that in addition to increased insulin resistance in peripheral tissues, augmented endogenous glucose production is also important to generate the higher hyperglycemia in 6-month-old obB1B2KO mice. This conclusion is supported by higher glucose production in the pyruvate challenge test and the increased hepatic mRNA expression of FoxO1, PEPCK, G6Pase, FbP1 and PGC-1a, whose protein products are linked with the activation of gluconeogenesis [33], [34]. Therefore, since insulin is known to decrease the expression of these genes in liver [34], these data also suggest that the absence of the kinin receptor can decrease hepatic insulin sensitivity in these animals. Hepatic endogenous glucose production is the main cause of hyperglycemia in human T2DM patients [5]. Thus it is tempting to speculate that the KKS may influence hepatic endogenous glucose production and the pathophysiology of T2DM in humans. In this case, the difficulty of achieving glycemic control in some patients may be related to different activity of the KKS in these patients.

ObB1B2KO mice also present increased hepatomegaly with more severe steatosis and augmented plasma concentration of ALT and AST in comparison to obWT mice. These changes appear although they display no differences in serum cholesterol and triglycerides levels. The worsening of steatosis in obB1B2KO mice suggests that KKS has a protective effect on the liver of these obese animals. The effects of double deletion of the B1 and B2 kinin receptors are related to enhanced renal injury in the Akita mice model of diabetes [35]. This observation fits well with findings of the present work, since we observed that the absence of kinin receptors also suggest a protective role of the KKS in these tissues. On the other hand, we have previously published that the absence of the B1 receptor is beneficial in lean mice lowering fasting glucose [15] and increasing the resistance to obesity induced by high fat diet [14]. It is important to note that resistance to obesity in these animals is dependent on increased leptin sensitivity and is therefore absent in mice additionally lacking leptin [14]. It is also known that in the absence of one of the kinin receptors, the expression of the other receptor increases in several tissues, a fact that can be related to changes in the phenotype of these models [11], [36].

The obese *ob/ob* mice have been used as a model for hepatic steatosis, although they normally present no inflammation. The mechanism involved in the development of steatosis in these animals is related to higher delivery of fatty acids to the liver and enhanced hepatic lipogenesis [37]. However, several studies show that leptin is essential for the deposition of hepatic fibrosis. Thus, *ob/ob* mice do not develop spontaneous or diet induced fibrosis [38], [39], [40], [41]. Previous work shows that bradykinin has a protective effect against liver damage and fibrosis induced in rats by treatment with carbon tetrachloride [42]. In this work, we provide evidences that the KKS is involved with hepatic metabolism, but we cannot show development of fibrosis due to the absence of leptin in our models.

In conclusion, we demonstrate that the KKS plays an important role in the pathophysiology of obesity and T2DM. Even though several reports describe the importance of the KKS in the pathophysiology of heart, kidney and retina diseases [12], [43], [44], little was known about the effect of the KKS upon the etiopathogenesis of obesity and T2DM, in particular in terms of liver function. This could pave the way for interventions that promote glycemic control in obese and T2DM patients.

## Materials and Methods

### Animals

Lep^ob^/Lep^ob^ and Bdkrb1.b2^tm^/Bdkrb1.b2^tm^ mice, referred in this work as obWT and B1B2^−/−^ respectively, all on C57BL/6j genetic background, were obtained from the Centre for the Development of Experimental Animal Models (CEDEME), Federal University of São Paulo (UNIFESP). The infertility of obWT male mice was reverted with white adipose tissue transplantation technique [45]. These males were used to mate B1B2^−/−^ females to generate the Lep^ob^.Bdkrb1.b2^tm^/Lep^ob^.Bdkrb1.b2^tm^, referred below as obB1B2KO. Mice received commercial rodent food and neutral pH water *ad libitum* (Nuvi Lab, Nuvital Paraná, Brazil) and were maintained under a 12-h light/12-h dark cycle under constant room temperature (22±1°C) and humidity (60±3%) conditions. The experiments were carried out with females. All procedures complied with the standards for the care and use of animals as stated in the guide for the care and use of laboratory animals (UNIFESP).

### Glycemic Analysis

Glycemic tests were performed in 3- and 6-month-old mice. The glucose tolerance test (GTT) and insulin tolerance test (ITT) were carried out in animals fasted for 12 hours. To avoid stress there was an interval of 7 days between tests. Glycemia was measured using a glucometer (Accu-Chek Advantage) measuring blood drops obtained from the tail vein. For GTT 1 g glucose per kg of body weight (BW) and for ITT 1 IU of insulin per kg BW was injected intraperitoneally.

To analyze insulin and C-peptide levels in serum after glucose load, 12 hours starved 6-month-old mice were anesthetized with 250 mg/kg Avertin (tribromoethanol). The animals were placed on a thermal plate to keep body temperature around 37°C. After 15 minutes of stabilization, we measured glycemia by a drop of blood from the tail and collected blood samples from the retroocular plexus. These procedures were repeated sequentially after 3, 6, 10, 15 and 30 minutes and the serum was separated by centrifugation and frozen until use. Insulin and C-peptide levels were measured using a Rat/Mouse Insulin ELISA Kit and Rat/Mouse C-Peptide 2 ELISA Kit (Millipore Corporation, cat n^o^ EZRMI-13K and EZRMCP2-21K, respectively).

### Pyruvate Challenge

Mice deprived of food for 24 h were injected intraperitoneally with sodium pyruvate (0.5 g/kg). Blood samples were collected from the tail vein immediately before and at various time points (0–60 min) after the pyruvate load.

### Tissue Extraction and Immunoprecipitation

Mice were anesthetized with sodium thiopental and analyzed 10–15 min later. As soon as anesthesia was assured by the loss of pedal and corneal reflexes, the abdominal cavity was opened, the portal vein exposed and 0.2 mL normal saline with or without insulin (2 µg) injected. Two minutes after the insulin injection, a fragment from the liver was removed, and 3 minutes later, muscle tissue was collected, minced coarsely, and homogenized immediately in extraction buffer containing protease and phosphatase inhibitors, as described elsewhere [46]. Extracts were then centrifuged at 15,000 rpm and 4°C for 40 min to remove insoluble material, and the supernatants were immunoprecipitated with anti-IR (sc-711, rabbit polyclonal, Santa Cruz Biotechnology) and protein A-Sepharose 6 MB (Pharmacia, Uppsala, Sweden).

### Protein Analysis by Immunoblotting

The precipitated proteins were treated with Laemmli sample buffer [47] containing 100 mM dithiothreitol and heated in a boiling water bath for 5 min, after which they were subjected to SDS-PAGE in a Bio-Rad miniature slab gel apparatus (Mini-Protean). Electrotransfer of proteins from the gel to nitrocellulose membrane was performed for 120 min at 120 V in a Bio-Rad Mini-Protean transfer apparatus. Nonspecific protein binding to the nitrocellulose was reduced by preincubating the filter for 2 h in blocking buffer (5% nonfat dry milk, 10 mm Tris, 150 mm NaCl, 0.02% Tween 20). The nitrocellulose blot was incubated with anti-phosphotyrosine antibody (sc-508, mouse monoclonal antibody, Santa Cruz Biotechnology) overnight at 4°C and then incubated with 125I-labeled protein A. The results were visualized by autoradiography on a preflashed Kodak XAR film. Band intensities were quantified by optical densitometry (Hoefer Scientific Instruments, San Francisco, CA, USA; model GS300).

### Morphometric Analysis

To determine the growth curve and the obesity development, body weight was measured weekly until the mice reached the age of 26 weeks. Thirty-week-old mice were sacrificed and their body weights as well as their organ weights were measured. Liver pieces were saved in buffered formalin or frozen in liquid nitrogen for further analysis. For histopathologic analysis, liver sections were stained with hematoxylin & eosin and Masson’s trichrome protocol. To analyze the steatosis, a pathologist classified the slides in a blind analysis in two classes of steatosis types (predominantly microvesicular or macrovesicular lesions) and in 4 classes for the levels of steatosis (0 to 25%, 26 to 50%, 51 to 75% and 76 to 100% of areas with steatotic lesions).

### Insulin Secretion in Isolated Islets

Pancreatic islets were isolated by collagenase digestion as described previously [48]. Briefly, the mice were sacrificed by cervical dislocation, and collagenase was administered into the pancreas by retrograde injection via the pancreatic duct. After 15–20 minutes of incubation at 37°C, the islet suspension was washed 4 times with Hank’s balanced salt solution at 4°C, before being manually collected. For static incubation, groups of four freshly isolated islets were initially pre-incubated for 45 min at 37 °C in Krebs-Ringer bicarbonate buffer with the following composition (in mmol/l): NaCl, 115; KCl, 5; CaCl_2_, 2.56; MgCl_2_, 1; NaHCO_3_, 24; and glucose, 5.6; supplemented with BSA (0.3% w:v) and equilibrated with a mixture of 95% O_2_∶5% CO_2_, pH 7.4. The solution was then replaced, and the islets incubated for 90 min under the experimental conditions (2.8, 5.6, 11.2, 16.8, 22.4, 28.0 or 33,6 mmol/l glucose). For dynamic incubations, groups of 50 freshly isolated islets were placed on Millipore SW 1300 filters (8.0 µm pore) and perifused with Krebs-Ringer bicarbonate buffer at a flow rate of 1 mL/min for 30 min in the presence of 2.8 mmol/L glucose (basal conditions). Subsequently, the islets were perifused with 11.1 mmol/L or 22.2 mmol/L of glucose. Perifusion solutions were gassed with 95% O_2_/5% CO_2_ and maintained at 37°C. Samples of perifused solution were collected each 1 or 2 min. Insulin release was measured by radioimmunoassay.

### Body Composition

Total body fat was estimated in 25-week-old mice by dual-energy X-ray absorptiometry using a Hologic QDR 4500 scanner (Hologic, Waltham, MA) as described previously [49].

### Gene Expression Analysis

Quantitative real-time PCR (qRT-PCR) was used to determine expression levels of distinct liver mRNAs. Briefly, tissue pieces from 12 hours fasted mice were removed, snap frozen in liquid nitrogen, and stored at −80°C until use. Samples were homogenized and total RNA was isolated using a NucleoSpin RNA II purification kit (Macherey-Nagel) and then stored at −80°C until use. The RNA integrity was assessed by electrophoresis on agarose gels. cDNA was synthesized from 1 µg of total RNA with Moloney murine leukemia virus reverse transcriptase (Promega) using random hexamer nucleotides. Standard curves for the primers were made to determine the amplification efficiencies of target and reference genes. Quantitative PCR was performed on an ABI Prism 7900 sequence detection system with 100 nM primers and 5 ng of cDNA. Target mRNA expression was normalized to β-actin expression and expressed as a relative value using the comparative threshold cycle (Ct) method (2^−ΔΔCt^) according to the manufacturer’s instructions. Expression levels from genes of interest were normalized to obWT control mice and presented as fold change. The primers sequences used are available in table S2.

### Statistical Analysis

All values were expressed as means + SEM. Statistical analyses were carried out using the two-tailed Student’s unpaired t-test to compare two independent groups or ANOVA followed by Bonferroni’s test to compare more than two groups. Contingency Table was used to analyze value distribution. Significance was rejected at P>0.05.

## Supporting Information

Table S1 **Mass of organs and tissues.**
(DOCX)

Table S2 **Primers sequences for qPCR.**
(DOCX)
