# Supplementary material for: Altered Glucose Homeostasis and Hepatic Function in Obese Mice Deficient for Both Kinin Receptor Genes
Source: PLoS One. 2012 Jul 19;7(7):e40573. doi: 10.1371/journal.pone.0040573 (PMC3400662; doi:10.1371/journal.pone.0040573)
Supplement: Table S1 — Mass of organs and tissues. (DOCX) [file pone.0040573.s001.docx]

Supplementary data

Table S1: Mass of organs and tissues.

|  | **obWT** | | |  | **obB1B2KO** | | | |
| --- | --- | --- | --- | --- | --- | --- | --- | --- |
|  | Average |  | S.E.M | n | Average |  | S.E.M | n |
| Body Mass (g) | 63,14 | + | 1,48 | 11 | 63,17 | + | 1,17 | 15 |
| Liver | 42,20 | + | 2,32 | 11 | 53,73 | + | 3,43 | 15 |
| Spleen | 1,74 | + | 0,33 | 11 | 2,26 | + | 0,22 | 15 |
| Kidneys | 6,59 | + | 0,27 | 11 | 6,16 | + | 0,14 | 15 |
| Triceps Sural | 3,76 | + | 0,18 | 11 | 3,73 | + | 0,16 | 13 |
| Heart | 2,79 | + | 0,10 | 11 | 2,56 | + | 0,06 | 15 |
| Left Ventricle | 1,80 | + | 0,07 | 11 | 1,68 | + | 0,04 | 14 |
| Right Ventricle | 0,52 | + | 0,03 | 11 | 0,50 | + | 0,04 | 15 |
| Atrium | 0,21 | + | 0,02 | 11 | 0,17 | + | 0,02 | 13 |
| Gonadal Adipose Tissue | 73,88 | + | 8,90 | 11 | 60,49 | + | 5,28 | 15 |
| Perirenal Adipose Tissue | 41,39 | + | 7,94 | 11 | 28,95 | + | 2,73 | 15 |
| Sub Cutaneous Adipose Tissue | 192,95 | + | 9,46 | 6 | 212,35 | + | 9,01 | 10 |

**Data of organs and tissues presented as mg per g body weight.**
