# Supplementary material for: Altered Glucose Homeostasis and Hepatic Function in Obese Mice Deficient for Both Kinin Receptor Genes
Source: PLoS One. 2012 Jul 19;7(7):e40573. doi: 10.1371/journal.pone.0040573 (PMC3400662; doi:10.1371/journal.pone.0040573)
Supplement: Table S2 — Primers sequences for qPCR. (DOCX) [file pone.0040573.s002.docx]

**Table S2:** Primers sequences for qPCR

| **GENE** |  | **Primer Sequence** |  |
| --- | --- | --- | --- |
| Beta actin | 5’- | TACAATGAGCTGCGTGTG | -3’ |
|  | 5’- | CACAGCCTGGATGGCTAC | -3’ |
| Glucose-6-phosphatase | 5’- | TCGGAGACTGGTTCAACCTC | -3’ |
|  | 5’- | ACAGGTGACAGGGAACTGCT | -3’ |
| Glucokinase | 5’- | GAGATGGATGTGGTGGCAAT | -3’ |
|  | 5’- | ACCAGCTCCACATTCTGCAT | -3’ |
| Carnitine Palmitoyltransferase 1 | 5’- | CTTCCATGACTCGGCTCTTC | -3’ |
|  | 5’- | AGCTTGAACCTCTGCTCTGC | -3’ |
| Stearoyl-CoA Desaturase-1 | 5’- | CCTGCGGATCTTCCTTATCA | -3’ |
|  | 5’- | GTCGGCGTGTGTTTCTGAG | -3’ |
| Hepatic Nuclear factor 4 | 5’- | CTCACCTCAGCAATGGACAG | -3’ |
|  | 5’- | GGCAGGAGCTTGTAGGATTC | -3’ |
| SREBP-1a | 5’- | GAACTGGACACAGCGGTTTT | -3’ |
|  | 5’- | GGCCAGAGAAGCAGAAGAGA | -3’ |
| Ppargc1a | 5’- | GTCAACAGCAAAAGCCACAA | -3’ |
|  | 5’- | TCTGGGGTCAGAGGAAGAGA | -3’ |
| Phosphoenolpyruvate carboxykinase | 5’- | CTAACTTGGCCATGATGAACC | -3’ |
|  | 5’- | CTTCACTGAGGTGCCAGGAG | -3’ |
| Fructose bisphosphatase 1 | 5’- | GACCCTGCCATCAATGAGTA | -3’ |
|  | 5’- | GTTGGCGGGGTATAAAAAGA | -3’ |
| TBP | 5’- | CCCTATCACTCCTGCCACACC | -3’ |
|  | 5’- | CGAAGTGCAATGGTCTTTAGGTC | -3’ |
| GAPDH | 5’- | GCTGTGGGCAAGGTCATCC | -3’ |
|  | 5’- | CTTCACCACCTTCTTGATGTC | -3’ |
| B2 bradykinin receptor | 5’- | CATCGAAATGTTCAACGTCAC | -3’ |
|  | 5’- | ATTGAGCCAACTCCACCACT | -3’ |
| Forkhead box protein O1 | 5’- | GCTTTTGTCACGATGGAGGT | -3’ |
|  | 5’- | CGCACAGAGCACTCCATAAA | -3’ |
| Forkhead box protein O3 | 5’- | TACGAGTGGATGGTGCGCTGTG | -3’ |
|  | 5’- | TCTTGCCCGTGCCTTCATTCTG | -3’ |
| Forkhead box protein O4 | 5’- | ACAGAATGCCTCAGGATCTGG | -3’ |
|  | 5’- | TGAAGTCCAGTCCCTCACCAT | -3’ |
| 25-hydroxycholesterol 7-alpha- | 5’- | TTGTAGCCCTCTTTCCTCCA | -3’ |
| hydroxylase | 5’- | GCTTGTTCCGAGTCCAAAAG | -3’ |
